# Proteomic dataset: Profiling of cultivated *Echerichia coli* isolates from Crohn's disease patients and healthy individuals

**DOI:** 10.1016/j.dib.2019.103734

**Published:** 2019-03-07

**Authors:** Daria Rakitina, Julia Baikova, Olga Pobeguts, Olga Bukato, Ivan Butenko, Irina Garanina, Mark Levites, Alexander Manolov, Alexandra Kanygina, Elena Kostryukova, Tatiana Semashko, Irina Karpova, Vladislav Babenko, Petr Scherbakov, Igor Khalif, Marina Shapina, Asfold Parfenov, Irina Ruchkina, Oleg Knyazev, Nina Fadeeva, Alexandr Subbotin, Sergey Chamkin, Alexandr Pyrkh, Marina Ivantsova, Vadim Govorun

**Affiliations:** a Federal Research and Clinical Center of Physical-Chemical Medicine of Federal Medical Biological Agency, Moscow, Russia; b Moscow Institute for Physics and Technology, Moscow, Russia; c Moscow Clinical Research Centre, Moscow, Russia; d State Scientific Center of Coloproctology, Ministry of Health of Russian Federation, Moscow, Russia; e City Clinic #13 of Nigjny Novgorod, Russia; f Poliklinika of Railways of Syktyvkar, Russia; g Clinic “Vivea” Khabarovsk, Russia; h S.V. Nudelman Medical Center of Plastic Surgery, Ekaterinburg, Russia

**Keywords:** *E. coli*, Proteome, Crohn's disease, HPLC-MS/MS

## Abstract

One of the dysbioses often observed in Crohn's disease (CD) patients is an increased abundance of *Escherichia coli* (10–100 fold compared to healthy individuals) (Gevers et al., 2014). The data reported is a large-scale proteome profile for *E. coli* isolates collected from CD patients and healthy individuals. 43 isolates were achieved from 30 CD patients (17 male, 12 female, median age 30) and 19 isolates from 7 healthy individuals (7 male, median age 19). Isolates were cultivated on LB medium at aerobic conditions up to medium log phase. Protein extraction was performed with sodium deoxycholate (DCNa) and urea, alcylation with tris(2-carboxyethyl)phosphine and iodacetamide. Protein trypsinolysis was performed as described in (Matyushkina et al., 2016). Total cell proteomes were analysed by shotgun proteomics with HPLC-MS/MS on a maXis qTOF mass-spectrometer. The data including HPLC-MS/MS raw files and exported Mascot search results was deposited to the PRIDE repository project accession: PXD010920, project https://doi.org/10.6019/PXD010920.

**Table:** Specifications table

Subject area	Biology
More specific subject area	Proteomics
Type of data	LC-MS/MS data and identification data
How data was acquired	maXis qTOF after the HDC-cell upgrade (Bruker, Germany) with a nano-electrospray source coupled to a Ultimate-3000 HPLC system (Thermo Scientific, USA).
Data format	Raw and analyzed data
Experimental factors	104 HPLC-MS/MS runs were performed: 65 samples for 43 isolates from 30 CD patients (17 male, 12 female, median age 30), 35 samples for 19 isolates from 7 healthy patients (7 male, median age 19), and 4 lab strains samples.
Experimental features	*E. coli* isolates collected from Crohn's disease patients and healthy individuals were cultivated on LB medium at aerobic conditions up to medium log phase and their total proteomes were analyzed by shotgun proteomics by HPLC-MS/MS.
Data source location	Research and Clinical Center of Physical-Chemical Medicine, Moscow, Russian Federation
Data accessibility	Data was deposited to the PRIDE repository: Project accession: PXD010920 Project https://doi.org/10.6019/PXD010920
Related research article	Bukato O, Garanina I, Matyshkina D, Pobeguts, O, Rakitina D, Baykova J, Ladygina V, Scherbakov P, Govorun V. (2017) Proteomic profiling of *E. coli*, isolated from Crohn's disease patients. FEBS JOURNAL, 284: SpT.5.3001, https://doi.org/10.1111/febs.141.

**Table:** 

**Value of the data** - The dataset contains the first published wide-range proteome analysis of Escherichia coli isolates from Crohn's disease patients and healthy individuals (104 raw HPLC-MS/MS analyses searched against three different databases) and is valuable for researchers interested in bacterial proteomics - The data can be of value for the studies of pathogenic/nonpathogenic Escherichia coli - The data might be useful in studies of Crohn's disease pathogenesis mechanism

## 1 Data

**Fig. 1:**
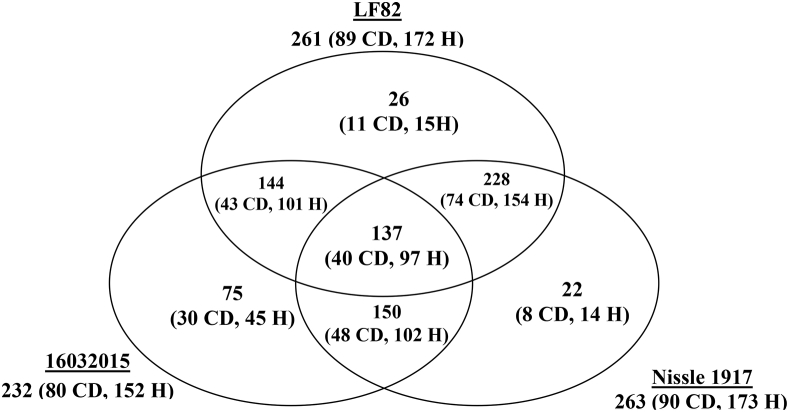
Number of common and unique proteins, significantly (p-value ≤ 0.05) overrepresented in Crohn's disease (CD) or healthy (H) isolates, identified during search against three databases.

**Fig. 2:**
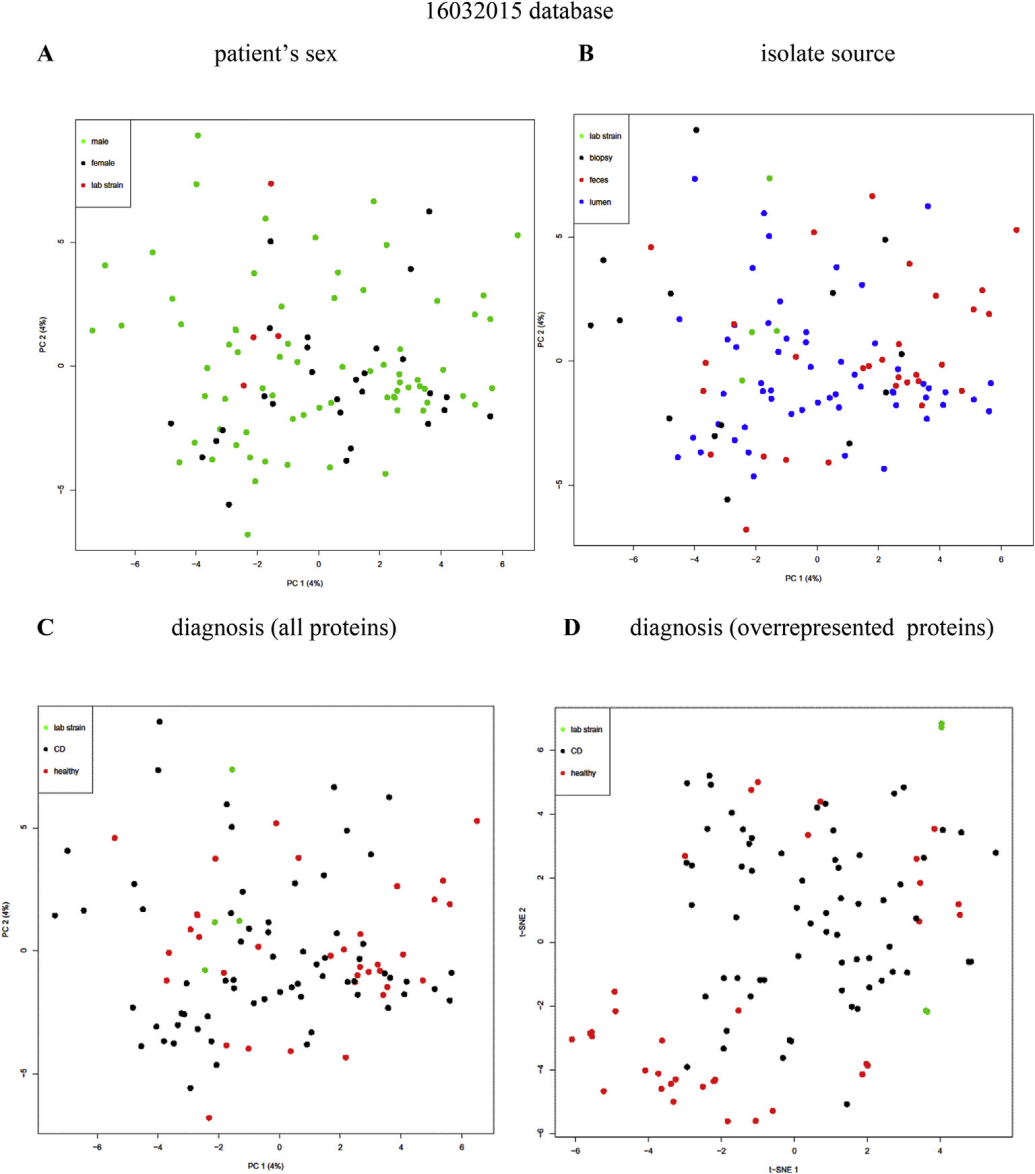
PCA clustering plot of proteins identified vs 16032015 database in proteomes of CD and healthy *E. coli* isolates. A, B, C, – all proteins, D – proteins significantly overrepresented in CD or healthy group. Various samples parameters are indicated. A – patient's sex (black dots – female, green – male, red – lab strains). B – isolate source (black dots – biopsy, blue – lumen, red – feces, green – lab strains). C, D – diagnosis (black dots – CD, red – healthy, green – lab strains).

**Fig. 3:**
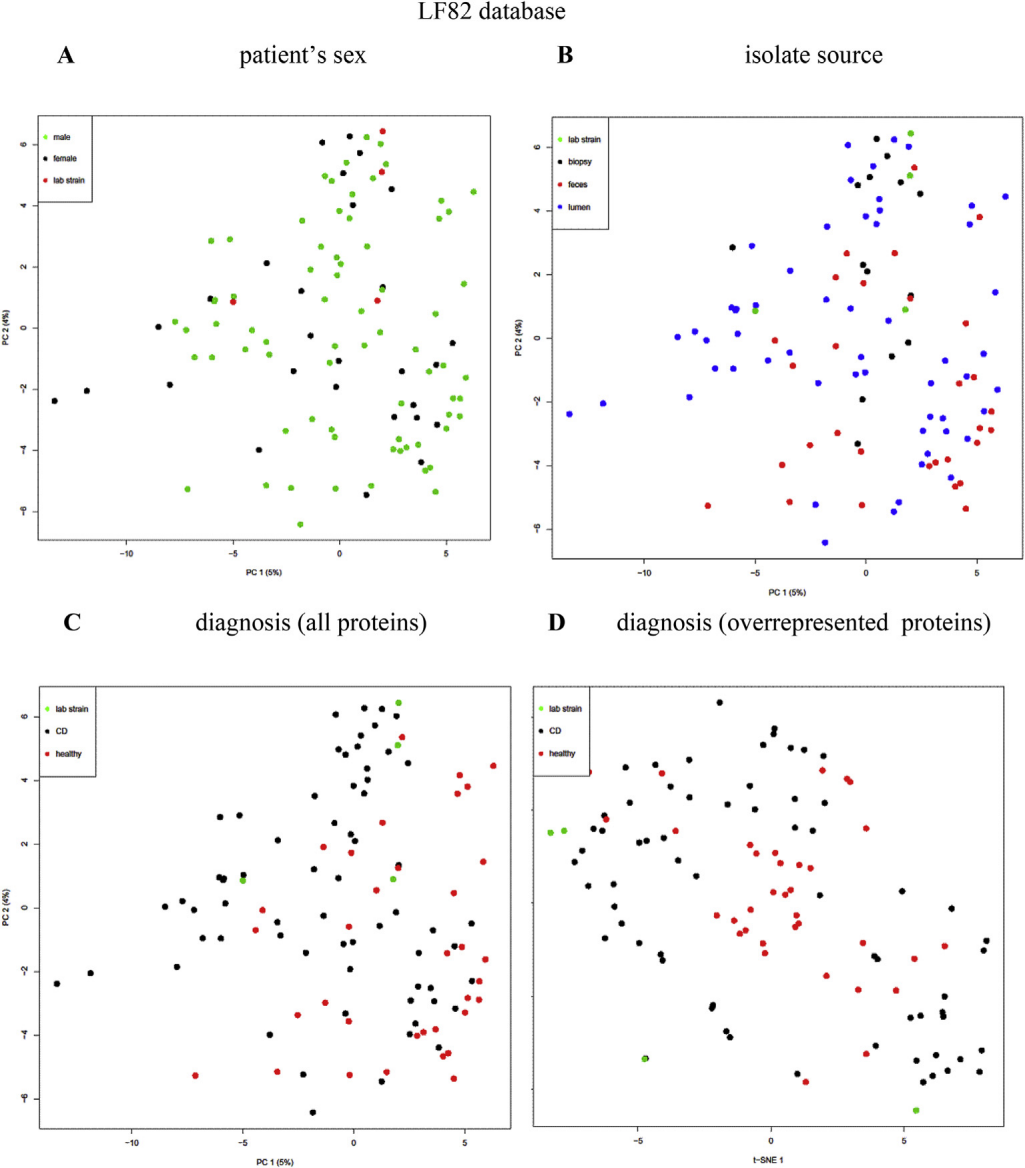
PCA clustering plot of proteins identified vs LF82 database in proteomes of CD and healthy *E. coli* isolates. A, B, C, – all proteins, D – proteins significantly overrepresented in CD or healthy group. Various samples parameters are indicated. A – patient's sex (black dots – female, green – male, red – lab strains). B – isolate source (black dots – biopsy, blue – lumen, red – feces, green – lab strains). C, D – diagnosis (black dots – CD, red – healthy, green – lab strains).

**Fig. 4:**
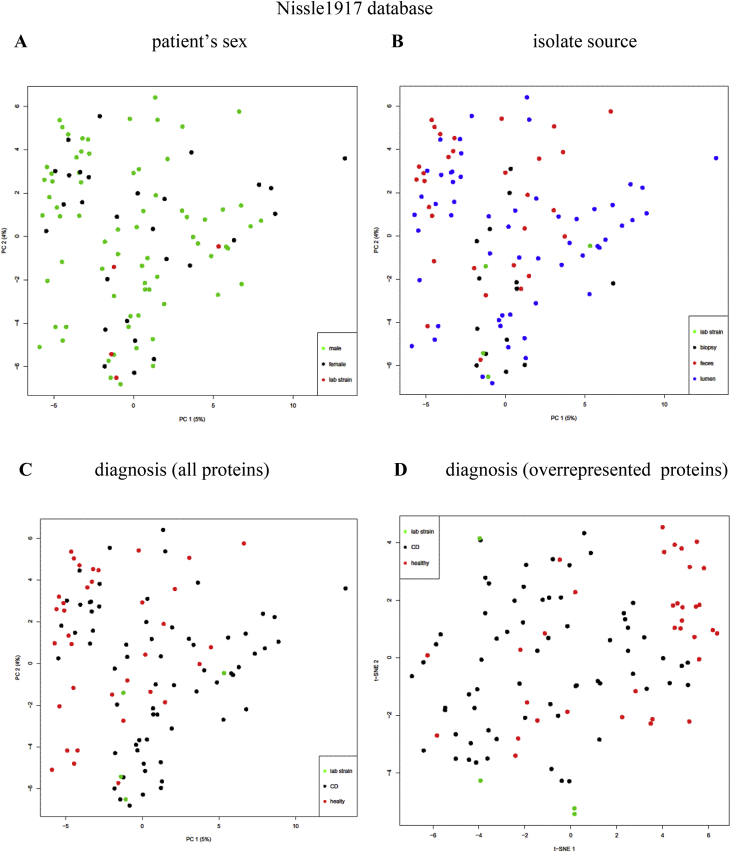
PCA clustering plot of proteins identified vs Nissle 1917 database in proteomes of CD and healthy *E. coli* isolates. A, B, C, – all proteins, D – proteins significantly overrepresented in CD or healthy group. Various samples parameters are indicated. A – patient's sex (black dots – female, green – male, red – lab strains). B – isolate source (black dots – biopsy, blue – lumen, red – feces, green – lab strains). C, D – diagnosis (black dots – CD, red – healthy, green – lab strains).

Escherichia coli is often observed as an abundant bacteria in intestines of Crohn's disease (CD) patients (Gevers et al., 2014) [1], in cotrast with healthy individuals. To identify proteins expressed in *E. coli* isolates from CD patients and healthy individuals (listed in Supplementary Table 1), we carried out HPLC-MS/MS proteome analysis of cultivated bacterial cells. Analyses were performed at maXis qTOF mass-spectrometer. Dataset covers 104 samples. Lists of identified proteins during search against three databases are given in Supplementary Tables 2, 3 and 4. Proteins, significantly overrepresented in CD or healthy isolates identified are listed in Supplementary table 5, and their functions are summarized in Supplementary Table 6 and Table 1. Numbers of proteins, significantly overrepresented in CD or healthy isolates, identified during search against three databases are given on Fig. 1. Principal component analysis (PCA) of *E. coli* proteomes with indication of patient's sex, isolate sources and diagnoses are given on Fig. 2, Fig. 3, Fig. 4.

**Table 1:** Functions of proteins, significantly overrepresented in CD or healthy isolates of *E. coli* (Summary table of functions of overrepresented proteins that are found in at least one database). Functions and number of proteins involved in each function are listed for CD-enriched and healthy-enriched proteins.

General functions	CD-overrepresented		healthy-overrepresented	
	function	number of proteins	function	number of proteins
antibiotic resistance	antibiotic resistance	2	antibiotic and metall resistance	3
respiration/oxidation	electron transfer	3	energy homeostasis and in adenine nucleotide metabolism	2
	anaerobic respiration	1	anaerobic respiration, response to DNA damage	1
	cell redox homeostasis	3	cell redox homeostasis	1
	Fe uptake	2	electron transfer	6
			oxidoreductase	5
			NAD(+) biosynthesis	1
			NAD-oxidoreductase, DNA damage stress	1
			Fe uptake	3
			protoporphyrin-IX biosynthesis	1
metabolic	carbohydrate metabolism	7	alcohol metabolism	7
	glycolytic process	1	Amino-sugar metabolism	1
	glyoxylate and dicarboxylate metabolism.	1	ATP biosynthesis	2
	indole production	1	carbohydrate metabolism	22
	alcohol metabolism	2	ethanol biosynthetic process	1
	lactate metabolism	1	fatty acids degradation	1
	propanoate metabolism	1	glutathione biosynthesis	1
	carbon utilization	1	glycolysis	1
	molybdopterin biosynthesis	1	IMP biosynthesis via de novo pathway	1
	mycothiol biosynthesis	1	isoprenoid biosynthetic process	1
			phosphonate metabolism	1
			polyol metabolism	4
			pyruvate metabolism	3
			Sulfur metabolism	1
			teichoic acid biosynthesis	1
			tricarboxylic acid cycle	2
capsule biosynthesis			biofilm formation	1
			capsule biosynthesis	1
			cell division	1
			cell envelope, cell wall biogenesis	6
			spore coat biogenesis	1
membrane biosynthesis	fatty acids metabolism, biosynthesis	1	lipid biosynthesis	1
	glycerolipid biosynthesis	1	lipopolysaccharide biosynthesis	1
	lipid biosynthesis	2	glycerophospholipid metabolism	1
	lipopolysaccharide biosynthesis	2	lipoprotein	6
	lipoprotein biosynthesis	1	outer membrane protein	3
membrane proteins	flagellin	1	outer membrane transporter	3
	outer membrane transporter	6	membrane protein	1
	inner membrane protein	3	molybdate ion transport	1
	antigen	1	quorum sensing	1
DNA	cell division	1	DNA	1
	DNA binding	2	DNA damage	2
	purine metabolism/biosynthesis	1	DNA recombination	4
	pyrimidine metabolism	2	DNA replication	1
			purine metabolism/biosynthesis	9
			pyrimidine metabolism/biosynthesis	5
			nucleotide metabolism	2
			nucleotide sugars metabolism	2
RNA	RNA degradation	1	transcription	4
	transcription	7	tRNA biosynthesis	4
			translation	2
			ribosomal	3
protein processing	peptidase	1	peptidase	4
	protein phosphorylation	2	protease	2
	protein transport	1	protein dephosphorylation	1
	proteolysis	1	protein folding	1
			protein kinase	1
			protein maturation	3
			protein secretion	1
			signal peptide processing	1
			enzymes activity regulation	1
translation	aa metabolism, biosynthesis, transport	7	aa biosynthesis	23
	translation	4	aa catabolism	1
	ribosomal	31	aa metabolism	4
	tRNA biosynthesis	5	aa transport	1
			cytosol protein	2
stress protection	chaperone	2	chaperone	3
	stress protein	8	stress	18
	Uncharacterized/hypothetical protein	6	Uncharacterized/hypothetical protein	9
vitamin, coenzyme, cofactor	vitamin transport, oxidation	1	vitamin biosynthesis	8
	coenzyme, cofactor biosynthesis	4	cofactor, coenzyme biosynthesis	3
virulence			virulence, host interaction protein	2

## 2 Experimental design, materials, and methods

### 2.1 Patients and samples

*Escherichia coli* isolates achieved from feces, ileum biopsy and liquid ileal content of Crohn's disease (CD) patients and ileal content and feces of healthy patients. Samples from CD patients were collected during diagnostic endoscopy at Central Scientific Institute of Gastroenterology (Moscow Clinical Research Centre, Moscow), State Scientific Center of Coloproctology (Ministry of Health of Russian Federation, Moscow) and Federal Research and Clinical Center of Physical-Chemical Medicine (Federal Medico-Biological Agency, Moscow). Duration of the disease was from four months to eight years. All patients had confirmed Crohn's disease three months before enrolment or earlier. The inclusion criteria were age above 18, endoscopically and radiologically diagnosed, and histologically confirmed Crohn's disease. The exclusion criteria were signs of indeterminate colitis, infectious diseases, anamnesis of total colectomy, presence of stoma, and recent antibiotic treatment.

Feces from healthy patients were collected in Clinical Center of Physical-Chemical Medicine (Federal Medico-Biological Agency, Moscow). Most material collections were performed in Moscow, however, some samples from CD patients were achieved by specialists of FRCCPCM during official visits to the hospitals in other regions of Russian Federation: Khabarovsk (2 patients), Syktyvkar (2 patients), Nizhny Novgorod (2 patients). Material collection was approved by local Ethics Committees, patients gave written informed consent for research and publication of data.

### 2.2 *E. coli* isolation and cultivation

Isolation of *E. coli* was as follows: liquid aspirates were diluted approximately ×10^6^ fold with sterile PBS. Approximately 0.05 ml volume of feces were placed into 0.5 ml of sterile PBS, vortexed to homogeneity, an aliquot was diluted approximately ×10^6^ fold. Biopsy samples were vortexed in 0.2 ml of sterile PBS. For all samples 0.1 ml of resulting liquid was spread onto LB agar plates. After overnight incubation on 37 °C, isolated colonies were identified as *Escherichia coli* on MALDI Mass-spectrometer Bruker Microflex with the MALDI Biotyper software (Bruker Daltonics, Germany) using the mass spectrometer Microflex LT (Bruker Daltonics, Germany).

Isolates were cultivated in LB at 37 °C (200 RPM) for 14 h. It was 3rd passage from the initial sample. Overnight cultures were diluted to 0.04 OD (540) and grown under the same conditions till mid-log phase (0.4 OD (540)). Bacterial cells were harvested by centrifugation (3500 g, 15 min) and pellet was washed twice with PBS.

### 2.3 Tryptic digestion of *E. coli* proteins

Protein trypsinolysis was performed as described in (Matyushkina et al., 2016) [2] with some alterations. Cell pellets were washed with PBS. Bacterial pellet was resuspended in 10 μl 100 mM NH4HCO3 with 0.5 mg/ml of lyzozyme and 1/10 volume of protease inhibitor mix. The suspension was incubated for 1 h at +4 °C. Then 10 μl of 10% of sodium deoxycholate (DCNa) and 1 μl nuclease mix (Promega) were added to the sample. The suspension was incubated for 1 h at +4 °C. Then the sample was diluted with 100 μl of 100 mM tris-HCl pH 8.0 with and 2.5mM EDTA. Cells were lyzed with ultrasonication for 1 min. Proteins were extracted with urea that was dissolved in each sample up to 6M concentration and incubated for 20 min at room temperature. After centrifugation for 10 min at 12 000 g, protein concentration was measured in supernatant by Bradford assay (Quick Start Bradford Protein Assay, BioRad) and samples were equalized.

The alcylation was performed as follows. 10 mM of reducing agent tris(2-carboxyethyl)phosphine (TCEP) was added and samples were incubated at 37 °C for 30 min. Then 30 mM of iodacetamide was added (IAA) and samples were kept at room temperature in the dark for 30 min. To avoid chemical modifications and remove the unreacted IAA, samples were treated with 5 mM TCEP and incubated for 20 min at RT. Protein hydrolysis was performed by trypsin (20 μg per sample, Trypsin Gold, Mass Spectrometry Grade, Promega) for 16 h at room temperature. After that samples were diluted with 6× volume of 100 mM tris-HCl pH 8.0 and protein hydrolysis was performed by addition of trypsin (in ratio trypsin : protein equal 1 : 50, Trypsin Gold, Mass Spectrometry Grade, Promega) in 0.1% SDS and incubation at 37 °C for 17h. At this point trypsinolysis stopped by addition of 10% TFA and incubation at 37 °C for 30 min. After centrifugation for 10 min at 12 000 g, supernatant was collected and cleaned with cartridges C18 (Discovery DSC-18 Tube, (Supelco)) according to the manufacturer's protocol. Achieved peptide extracts were dried at SpeedVac (Labconco) and dissolved in 15 μl of LC-MS-MS sample buffer containing 3% acetonitrile and 0.1% trifluoracetic acid. The equivalent of 5 μg of protein was loaded onto HPLC-MS/MS analysis.

### 2.4 HPLC-MS/MS analysis

The HPLC-MS/MS analysis of the tryptic peptides was carried out using an Ultimate-3000 HPLC system (Thermo Scientific) coupled to a maXis qTOF after the HDC-cell upgrade (Bruker) with a nano-electrospray source. The chromatographic separation of the peptides was performed on a trap-elute system: trap column (Zorbax 300SB-C18, 5 mm × 0.3 mm, particle diameter 5 μm, Dionex) and column (Zorbax 300SB-C18, 150 mm × 75 μm, particle diameter 3.5 μm, Agilent). The gradient parameters were as follows: 5–35% acetonitrile in aqueous 0.1% (v/v) formic acid, the column flow 0.3 μl/min. The gradient duration was 120 min. The positive MS and MS/MS spectra were acquired using an AutoMSMS mode (the capillary voltage 1700, the curtain gas flow is 4 l and the temperature is 170 °C, the spectra rate 4 Hz, 20 precursors, m/z range 200–1500, the active exclusion after 2 spectra, release after 0.5 min). The lists of compounds (mgf files) were generated after a lock mass calibration (m/z 445.1200) with a Compass DataAnalysis (Bruker).

### 2.5 Protein identification and quantitative analysis

The protein identification was performed by the peptide search with a Mascot Data Search with the following parameters: Peptide Mass Tolerance 0,05 Da, Fragment Mass Tolerance 0,1 Da, variable modifications Carbamidomethyl (C), Oxidation (M), cutting enzyme trypsin, 1 missed cleavage per peptide was allowed.

Peptide search for protein identification was performed versus database of proteins (peptides).

Databases for protein search by Mascot search were created as follows:

Ecoli-16032016-kerat.fasta - was created by translation and annotation by PROKKA 1.7 of 14 CD *E. coli* isolates and 12 isolates from healthy patients (summarized and described in Rakitina et al., 2017 [3]). Similar proteins (>80% homology at >80% sequence) were united and the one showing maximum similarity with the other group members was used as representative. The database included: total sequences 92600, total residues 32006615. The cut-off ion score was >28 as an indicator of identity (p-value <0.05).

Nissle1917_goodProt_kerat.fasta – was formed on the basis of genomes of genome of typical symbiotic *E. coli* strain).

Escherichia_coli_LF82_uid161965-1.fasta – was formed on the basis of genomes of genome of typical CD *E. coli* strain).

Aminoacid sequences of trypsin (Promega) and Human keratins were added to all databases to avoid misinterpretation of contaminating proteins. The protein was considered as identified by no less than two unique peptides with the score above the threshold. Lists of identified proteins are given in Supplementary Tables 2, 3 and 4.

The protein abundances were evaluated by a label-free method using an emPAI (Exponentially Modified Protein Abundance Index) determined by Mascot Data Search for each identified protein (Shinoda et al., 2010) [4]. Proteins significantly overrepresented in CD or healthy group are listed in Supplementary table 5. Numbers of proteins, significantly overrepresented in CD or healthy isolates, identified during search against three databases are given on Fig. 1.

### 2.6 Proteins abundance comparison between CD and healthy groups of *E. coli* isolates

The data of over- or under-represented proteins in CD and healthy groups of *E. coli* isolates, was achieved by the two-way Fisher test was used separately for each protein.

Principal component analysis (PCA) and T-distributed Stochastic Neighbor Embedding (T-SNE) analysis were used for data analysis. Principal components were constructed, representing orthogonal transformation of the analyzed data set. The principal component plot showed directions along which variation of data was maximum, so the 2d plot we can see the projection of distances among variables in multidimensional space. Variables in the 2d plot can group in clusters reflecting the correlation among variables like in clustering analysis. The test was performed in R with prcomp. T-SNE is a machine learning algorithm for visualization of high-dimensional data based on nonlinear dimensionality reduction. T-SNE analysis was performed in R with Rtsne.

Plotted 2D projections are given on Fig. 2, Fig. 3, Fig. 4. Patient's sex, isolate sources and diagnoses are indicated.
